# Recent discoveries on the structure of iodine(iii) reagents and their use in cross-nucleophile coupling

**DOI:** 10.1039/d0sc03266b

**Published:** 2021-01-07

**Authors:** Adriano Bauer, Nuno Maulide

**Affiliations:** Institute of Organic Chemistry, University of Vienna Währinger Strasse 38 1090 Vienna Austria maulide@univie.ac.at http://maulide.univie.ac.at

## Abstract

This perspective article discusses structural features of iodine(iii) compounds as a prelude to presenting their use as umpolung reagents, in particular as pertains to their ability to promote the selective coupling of two nucleophilic species *via* 2e^−^ oxidation.

## Abstract 2

This perspective article discusses structural features of iodine(iii) compounds as a prelude to presenting their use as umpolung reagents, to promote the selective coupling of two nucleophilic species *via* 2e^−^ oxidation.

## Graphical abstract

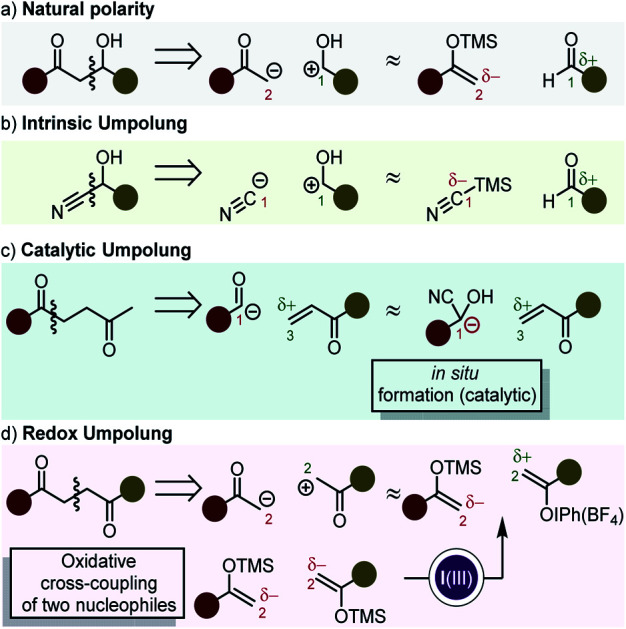

## Introduction

**Scheme 1:**
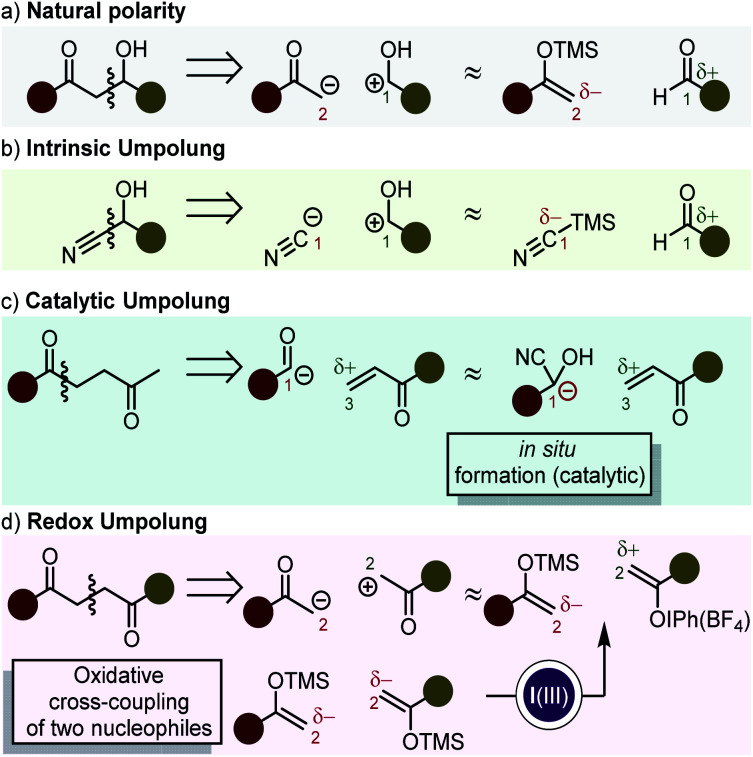
Retrosynthetic analysis and synthons in (a) a disconnection following natural polarity. (b) The cyanide anion as the canonical umpolung synthon. (c) Umpolung disconnection by translation of the acyl anion synthon into a catalytic species of the Stetter reaction. (d) Umpolung disconnection by the use of redox processes.

One of the cornerstones of modern organic synthesis was set with the advent of retrosynthetic analysis as a powerful systematic tool for planning a synthetic route.^1^ Its introduction made it increasingly more evident, that 1,3- or 1,5-heteroatom-substituted carbon frameworks are usually easily accessible because their synthons adhere to what is referred to as natural polarity (Scheme 1a). On the other hand, 1,2- or 1,4-heteroatom-substituted organic molecules present challenges resulting from the need that one of the synthons reacts with inversed polarity (*i.e.* umpolung).^2,3^ While some functional groups (*e.g.* cyanides, epoxides, deprotonated 1,3-dithianes^3,4^) display innate inversed polarity (intrinsic umpolung – Scheme 1b), other functionalities can formally switch their polarity by derivatization in a catalytic cycle (*e.g.* benzoin condensation, Stetter reaction – Scheme 1c).^5,6^

A third possibility is the implementation of (external) redox processes to reverse the polarity of a given functional group. This has been studied by the use of 1e^−^^7,8^ and 2e^−^ transfer reactions.

During the last three decades, iodine(iii) compounds have gained considerable attention in this regard as they are abundant, safe, environmentally benign and mild 2e^−^-oxidants with a broad application spectrum. They can be used as organocatalysts, by using a sacrificial oxidant^9–13^ or anodic oxidation.^13–15^ Their use in flow chemistry has been recently disclosed.^14^ The chemistry of iodine(iii) compounds is incredibly versatile and has been extensively reviewed.^9–11,13,15–37^ This minireview/perspective article will first discuss recent results regarding the structural features of iodine(iii) compounds and then focus on their use as umpolung reagents.

More specifically, their ability to promote the selective coupling of two nucleophilic species *via* 2e^−^ oxidation will be discussed.

Reactions which follow a redox umpolung approach (Scheme 1d) can be termed “cross-nucleophile couplings” in analogy to the term “cross-electrophile couplings” which refers on the use of two electrophilic species in combination with a reductant.^38^

## General aspects of the chemistry of organoiodine compounds

The chemical properties of iodine are determined by its relatively low electronegativity and its high polarizability compared to the lighter halogen elements. It is the heaviest non-metallic element with stable isotopes. In nature, iodine is mostly found as iodide and forms weak bonds with organic molecules, due to weak orbital interactions (*e.g.* the bond dissociation energy (BDE) of the C–I bond in H_3_C–I is only 55.9 kcal mol^−1^, almost only half as strong as the H_3_C–H bond (104.9 kcal mol^−1^)).^39^ Iodine and carbon have comparable electronegativities,^40^ by convention, iodine is considered less electronegative than carbon in iodanes (although the most commonly used electronegativity scales show the opposite relationship), so that PhI is considered an iodine(i) compound. This has no direct relevance for the reactivity, since oxidation states are formalisms (especially in organic molecules). We will follow this convention in the further discussion. In this regard, it might be important to remember that heavier p-block elements (*n* > 2 – including iodine) have a low tendency to form hybridized orbitals (sp^*x*^), because the higher s and the p orbitals do not have a comparable radial extent (they are different in “size”).^41–43^ Consequently, they form bonds by using orbitals with large p-character, while the s-orbital mostly takes the role of the lone pair (“inert pair effect”).^43^ When fully oxidized however, they are forced to use the s-orbital for bonding, which has dramatic effects on the chemical behavior, especially when the heavy atom is attached to electronegative elements.^43^

## Structure of iodine(iii) compounds

### Valence bond- and molecular orbital-picture

**Scheme 2:**
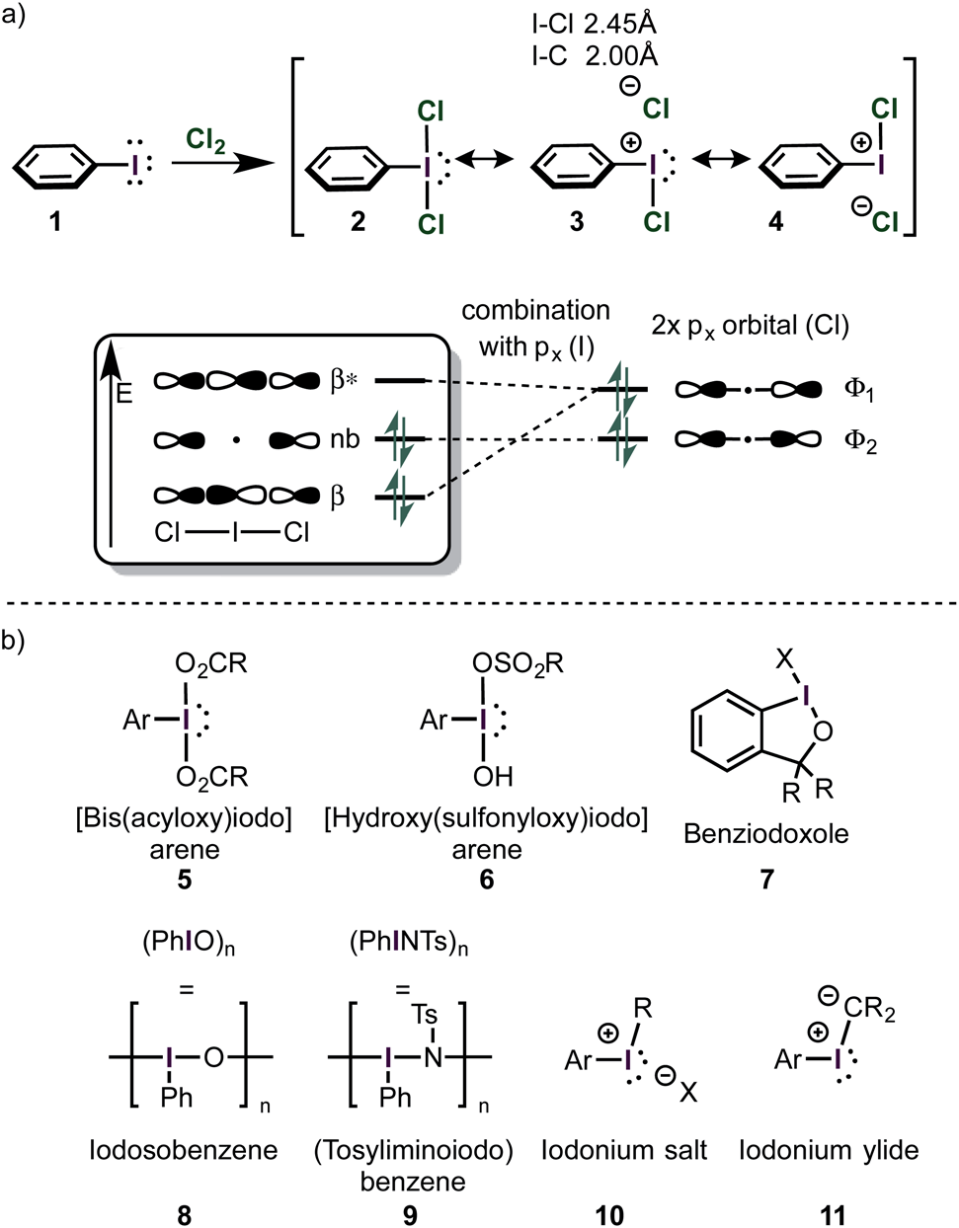
(a) The first synthesis of a λ^3^-iodane. The bonding situation can be described accurately by the VB (left) and the MO model (right). (b) Typical iodine(iii) compounds.

Because of iodine's low electronegativity, organoiodine(i) compounds can be oxidized relatively easily. The first isolated organoiodine compound with a higher oxidation state than (I) was the T-shaped (dichloroiodo)benzene (**2**) in 1885 (Scheme 2a).^44^ Neutral tricoordinated iodine(iii) molecules like **2** are called λ^3^-iodanes, whereas positively charged iodine(iii) compounds are called iodonium salts (**10** – Scheme 2b).^16,20^ λ^3^-Iodanes seem to violate the octet-rule because they are drawn with more than 8 electrons in their outer shell. Thus, they are often described as “hypervalent” species. It has been argued that the term is misleading because the octet rule is still satisfied in most systems.^41,43,45–48^ This goes along with the conclusion that empty d(*n* + 1)-orbitals of a given element are usually too high in energy to be accessed.^49,50^ However, it was demonstrated that subtle hypervalency and d-orbital participation in bonding (to a small but significant extent) has to be expected in some fully oxidized systems (*e.g.* SF_6_).^43,51,52^ In spite of this controversy the term “hypervalent” is, overwhelmingly accepted in the recent literature.

The bonding situation in neutral I(iii) compounds is accurately described as an ionic pair in two mesomeric forms in the valence bond model (Scheme 2a).^43^ Some iodine(iii) and iodine(v) compounds (including PhI(OAc)_2_, PhIF_2_, DMP and IBX) are actually hypovalent with regard to the iodine atom.^48^ By computational analysis of the valence electrons, it has been shown that the iodine atom in PhI(OAc)_2_ has 2 lone pairs, but only one binding orbital (to the phenyl moiety). The remaining two electron-pairs reside closely to the electron-withdrawing ligands, confirming the charged resonance structures **3** and **4**. This is in accordance with the unusual strong polarization of the I–OAc bond and explains the preference of the two axial ligands to be electron-withdrawing.

The equivalent commonly accepted molecular orbital picture combines the two p_*x*_ orbitals of Cl^−^ according to the *C*_2v_ symmetry of PhICl_2_. One of those combined orbitals (*Φ*_1_) has the right symmetry to interact with the p_*x*_ orbital of the iodine atom in PhI^2+^ to form a filled bonding (*β*) and an empty antibonding orbital (β*). The orbital *Φ*_2_ does not have the appropriate symmetry for an interaction and remains unaltered (Scheme 2a).^53–56^ This model is also in accordance with the ionic mesomers **3** and **4**, because the two electrons of the non-bonding orbital are mostly localized on the chlorine atoms, however the hypovalency of those species is not directly explained by this model.^43^

### σ-Holes and halogen bonding

**Scheme 3:**
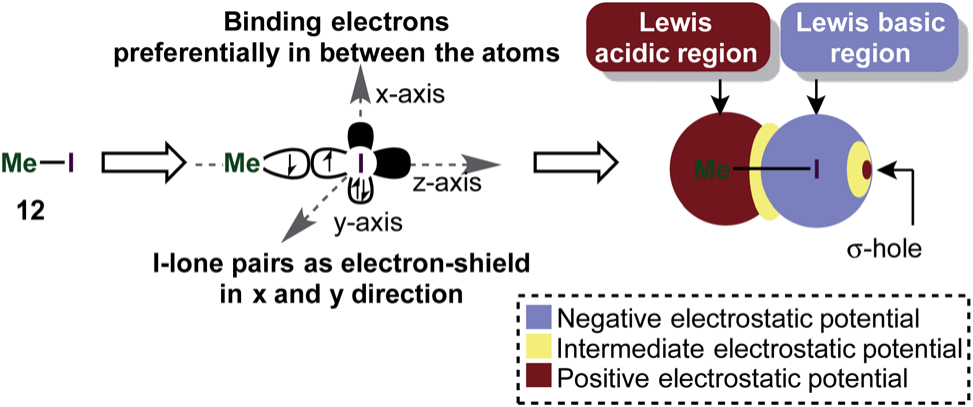
The approximate molecular electrostatic potential (MEP) shows the σ-hole in iodomethane.

Iodine(iii) compounds usually react as electrophilic oxidants. An important aspect of this reactivity is the so-called σ-hole, which often occurs in main-group elements and plays a key role in halogen bonding.^57^ A σ-hole is a region of positive electrostatic potential, which is caused by a σ-bond. Iodine in H_3_C–I for instance, is bonded *via* a half-filled p_*z*_-atomic orbital to CH_3_ and its electrons will be localized mostly in the interatomic region in order to build up the single bond (Scheme 3). This causes a depletion in electron density at the outer lobe on the *z*-axis. At the same time, the lone pairs in *x* and *y* directions are doubly filled and act as a shield of high negative electrostatic potential.^57^ The existence of a σ-hole influences not only on the reactivity, but also the association of molecules in the solid phase (*e.g.* H_3_C–I).^58^

This phenomenon is especially intriguing because it contradicts the common approximation that an atom in a molecule has a net atomic charge (*e.g.* by assigning a “δ−” to an atom). Generally speaking, the electrostatic potential of an atom in a molecule (the “charge”) is an anisotropic quantity, *i.e.* it is dependent on the physical location and can be usually illustrated as a surface around the atom on the van-der-Waals radius.^57^

**Scheme 4:**
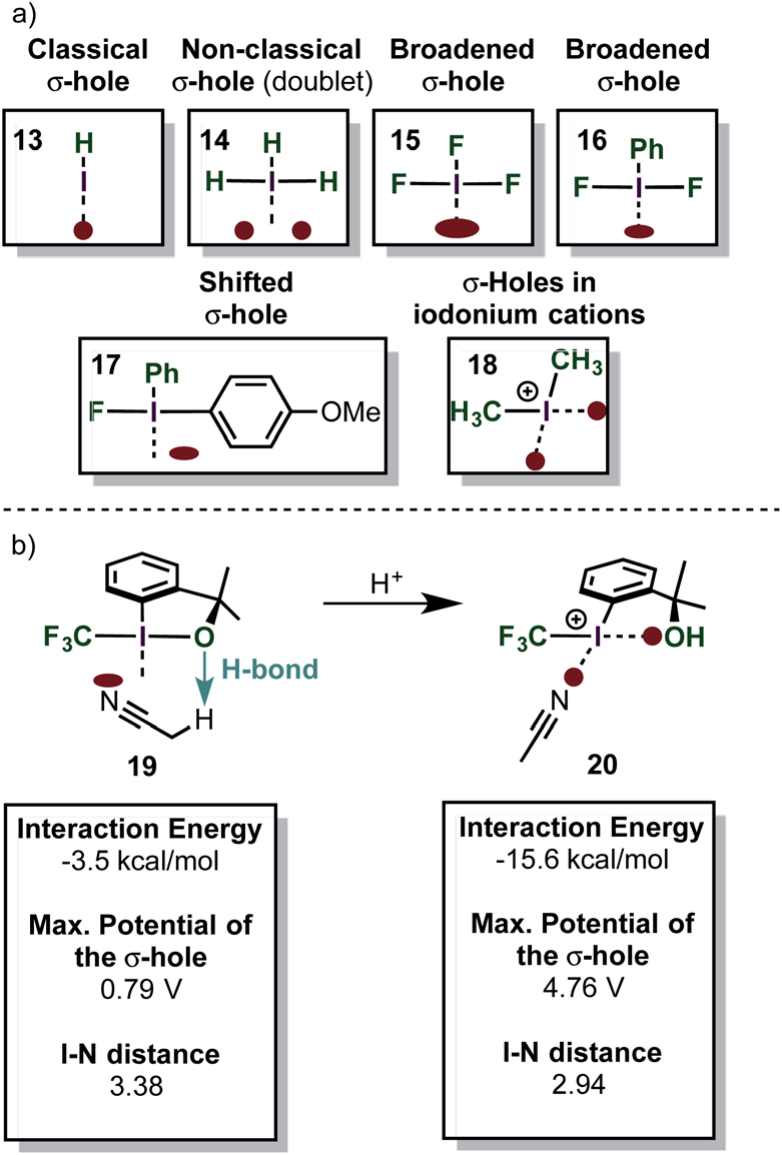
(a) σ-Holes in different iodine compounds. (b) Interaction of a non-classical σ-hole with acetonitrile.

The σ-hole in iodine(iii) species is caused by the classical 2-center-2-electron bond between the equatorial ligand and the iodine atom. The nature of this bond determines the strength of the σ-hole. Interestingly, the orthogonal 3-center-4-electron bond perturbs the shape of the σ-hole, so that two distinct maxima of positive electrostatic potential are observed (H_3_I) (*i.e.* the σ-hole forms a doublet – Scheme 4a).^59^ This phenomenon was termed a “non-classical σ-hole”. The coupling seems to be more pronounced when electron-neutral ligands are attached (IH_3_), while strongly electronegative atoms such as fluorine (IF_3_) make the σ-hole just appear broader. This broadening however is in fact caused by the appearance of two distinct maxima. When the two axial ligands are different, the σ-hole becomes an apparent singlet again, but is shifted towards one of the ligands. Iodonium cations, which are bound to the ligands by two classical 2-center-2-electron bonds, have two distinct classical σ-holes along the prolonged axes of the C–I-bond. This is in accordance with recent findings on halogen bonding to cyclic iodonium salts.^60^ Importantly, electron-rich species (*e.g.* nucleophiles) will form coordinative bonds to the σ-hole (σ-hole bonding).^59^ Acetonitrile, for example, was calculated to interact with the shifted σ-hole of Togni's reagent to form the adduct **19** (Scheme 4b). The I–N distance (3.38 Å) is typical for such an interaction. The non-classical σ-hole may also explain why the iodine atom in PhI(O_2_CR)_2_ is usually coordinated to 5 atoms in a planar pentagonal arrangement.^61,62^

λ^3^-Iodanes can be activated (*i.e.* they become more electrophilic) in acidic media if one of the ligands has sufficient basicity (PhI(O_2_CCF_3_)_2_ for instance cannot be activated by BF_3_·OEt_2_).^63^

After protonation, one of the ligands is detached from the iodine atom so that an iodonium species is formed (**20** – Scheme 4b). The former ligand, however, remains in relatively close proximity due its coordination to the new σ-hole in the iodonium species.

Moreover, the strength of the σ-hole *trans* to the phenyl ring is enhanced significantly and consequently the interaction energy of the iodine compound with the ligand (MeCN) gets stronger. Although the σ-hole interaction and its influence on the reactivity of iodine(iii) compounds has to be yet proven by experimental data, these results are in accordance with the fact that activated iodine(iii) compounds such as PhI(OH)(OTs)·HOTs have been described as Lewis acids.^64^ The Lewis acidity of iodonium salts has been studied and quantified very recently.^65^ In addition, X-ray crystal structures of several activated iodine(iii) compounds have been interpreted as iodonium species with a weak coordination to the activated ligand.^63^ The same article provides also a nice comparison of the LUMO energies of different activated iodine(iii) compounds. The relationship between molecular orbitals and σ-holes is not always obvious: while the isolated molecular orbitals are just artificial models which try to describe the reality under certain approximations and assumptions, the electron density around a given molecule is a real physical entity. The electron density with all its peculiarities (*e.g.* σ-holes) can be interpreted as the manifestation of the complex interplay of all orbitals of a certain species. Hohenberg and Kohn were the first to recognize this important relationship, which is also the basis of density functional theory (DFT) calculations.

The structure–reactivity relationship of the σ-holes in iodine(iii) compounds has yet to be investigated in depth. However, the previously described physico-chemical properties already explain the highly electrophilic/Lewis acidic behaviour of iodine(iii) compounds. It also rationalises the “bow tie” structure found in PhI(OAc)_2_ where both carbonyl functionalities of the carbonyl groups coordinate to the iodine atom.^16,66,67^ We hope that this perspective article will encourage scientists to further explore other peculiarities of iodine(iii) reactivity which are closely tied to the described structural features. The next section will showcase how iodine(iii) chemistry can be synergistically leveraged into the oxidative coupling of two nucleophilic species.

## Cross-nucleophile coupling using iodine(iii)

### General perspective

**Scheme 5:**
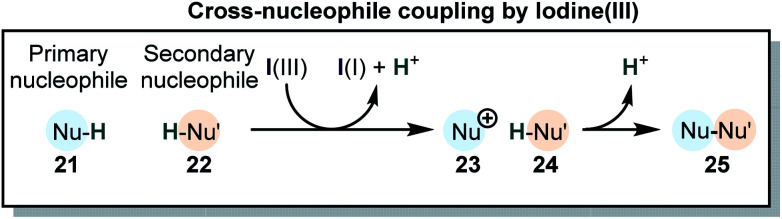
General picture of the cross-nucleophile coupling promoted by I(iii).

Many reactions which utilize iodine(iii) compounds can be categorized as cross coupling of two nucleophilic species by oxidation of one of the partners (Scheme 5). This type of reaction can be called “cross-nucleophile coupling” (in analogy to the “cross-electrophile coupling” in metal catalysis).^38^ The coupling is accompanied by the formal loss of two protons or equivalent electrofuges (*e.g.* TMS^+^, *t*Bu^+^). In some cases, the second nucleophile is attached to the iodine(iii) species, with loss of the nucleofuge prior to the reaction. These group-transfer reactions may be considered stepwise cross-nucleophile couplings.^28^ The overall reaction can be accompanied by cationic rearrangements in one of the two or both coupling partners. Interestingly, these general transformations are not only accessible *via* iodine(iii) compounds but can be also accomplished with Tl(iii) or Pb(iv). Indeed, the reactivities of iodine(iii), thallium(iii) and lead(iv) are closely related.^68–76^ The chemistry of Hg(ii)^76,77^ and Se(iv)^78^ is also akin to iodine(iii) compounds. However, due to their high toxicity and their environmental impact, Pb(iv) and especially Tl(iii) are seldom used in modern synthesis, because iodine(iii) compounds are much safer, readily tunable and environmentally benign.

### Cross-nucleophile coupling of phenols, anilines and amides

**Scheme 6:**
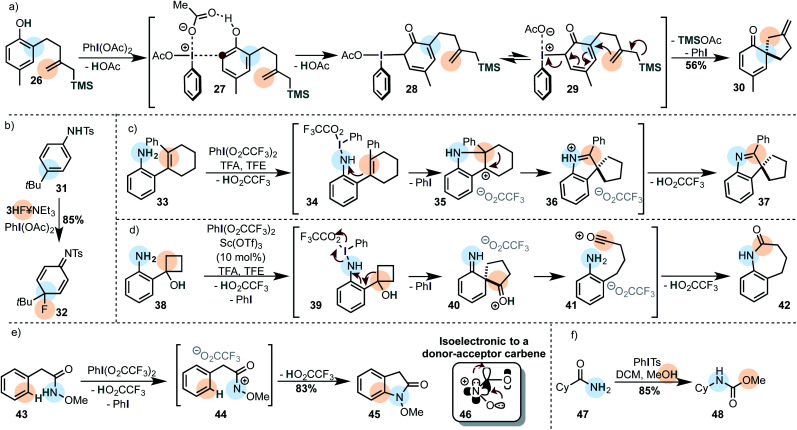
(a) Intramolecular cross-nucleophile coupling of a phenol with an allyl silane. (b) Cross-nucleophile coupling of a secondary aniline derivative with a fluoride. (c and d) Rearrangements on primary anilines induced by iodine(iii). (e) Intramolecular cross-nucleophile coupling of an arene with an amide promoted by I(iii). (f) Hoffmann-type rearrangement of an amide promoted by iodine(iii).

Phenols are typical substrates of cross-nucleophile coupling reactions and can be coupled to a variety of internal and external nucleophiles to furnish a dearomatized product. An interesting example is showcased in Scheme 6a.^79^

Phenols were classically proposed to coordinate to iodine(iii) *via* the oxygen-atom to form an electrophilic adduct.^80^ The intermediate is then intra- or intermolecularly trapped by a second nucleophile.^81^ A recent study suggests that the iodine(iii) is most likely attacked by the carbon atom of the phenol to form a C–I bond in the first step (*cf.***28**). This may either be formed in a concerted mechanism or by an isomerization of the iodine(iii) species with a subsequent coordination (*cf.***27**) probably involving the iodine's σ-hole. A second *cis*/*trans* isomerization then leads to the attack of the nucleophile through a dissociative (S_N_1-type) or an associative (S_N_2-type) mechanisms depending on the conditions.^82^ Our interpretation of the mechanism depicted in Scheme 6a is based on those findings. Especially the solvent has a large influence with dramatic effects on the stereoselectivity.^83,84^ Analogous reactions, where the iodine species is used catalytically with a secondary oxidant are known,^85,86^ including enantioselective versions.^83,84,87,88^

Similar reactions have been widely applied to secondary anilines and their derivatives (Scheme 6b),^89^ although the products usually rearomatize by the loss of an electrofuge (*e.g. t*Bu^+^, H^+^).^19^ Primary anilines on the other hand can be coupled intramolecularly to alkenes as the secondary nucleophiles (Scheme 6c).^90^ After attack of the double bond onto the umpolung species, the alkyl substituent in intermediate **35** migrates preferentially. However, in other cases the phenyl has a larger migratory aptitude to shift onto the carbocationic position. Not only alkenes were used as secondary nucleophiles in this study, but also strained cycloalcohols such as **38** which underwent a semi-pinacol rearrangement (Scheme 6d). If we assume an associative mechanism, where the iodine atom coordinates to the nitrogen of the aniline, it is likely that the breaking C–C bond is anti-periplanar to the breaking N–I bond. Alternative mechanisms have been proposed in which a nitrene is formed.

Carboxamides are also popularly used as oxidizable nucleophiles, especially *O*-methyl hydroxamic acids (Scheme 6e).^91^ The reaction mechanism involves the formation of an electrophilic nitrenium ion (**46**), which is isoelectronic to a donor–acceptor carbene. The structural similarity is also showcased by the fact that unstabilized acylnitrenium ions generated by iodine(iii) often rearrange to isocyanates (Scheme 6f – Hoffmann-type rearrangement),^92^ and undergo C–H insertions,^93^ reminiscent of nitrene/carbene reactivity. Indeed, the intermediate **44** may undergo either a classical Friedel–Crafts-type mechanism with the arene, or a C–H insertion reaction to yield the observed product. The nitrenium ion can be attached to a variety of nucleophiles, although the majority of the reports involves arenes, alkenes and alkynes.^19^ Some creative transformations have been developed using this approach, for instance a catalytic oxidative annulation of benzamides with alkynes.^94^††Importantly, this approach is not limited to the oxidation of carboxamides, but can be similarly applied to nitrogen atoms in benzimidazoles.^162^ α-Tertiary amines may undergo a 1,2-alkyl shift onto the nitrenium species. The thus generated iminium ion can be subsequently trapped by sodium cyanoborohydride as the secondary nucleophile.^95^

### Cross-nucleophile coupling involving simple ketones

**Scheme 7:**
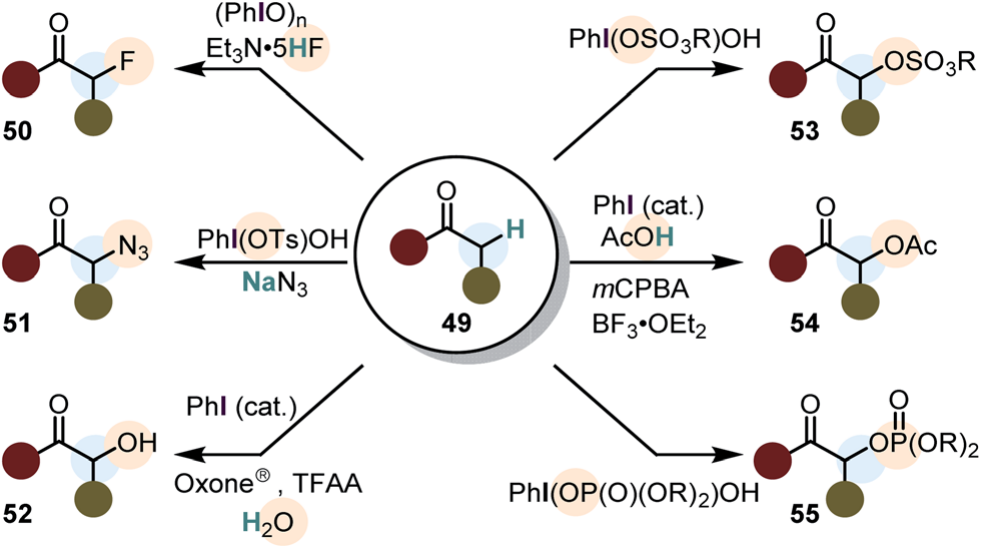
α-Functionalization of ketones using iodine(iii) compounds.

The α-functionalization of ketones (and their derivatives) represents arguably one of the most powerful transformations induced by iodine(iii) compounds (Scheme 7).^23,24^ It was already pioneered in the late 1960's,^96^ but Mizukami *et al.* were the first to describe the α-acetoxylation of simple ketones (acetophenones) with PhI(OAc)_2_ in 1978. The reaction was generally low yielding and a mixture of acetic acid and acetic anhydride had to be used as the solvent.^97^ However, in the last decade major developments in the direct α-functionalization of ketones were accomplished, including hydroxylations,^98–101^ tosyloxylations,^102–106^ mesyloxylations,^107^ phosphoryloxylations,^108,109^ fluorinations,^110^ azidations^111–113^ and acetoxylations.^114^ Some of these transformations are catalytic in iodine(iii)^100,103,104,106,114,115^ and enantioselective versions are known.^101,104–106,115^ However, intermolecular enantioselective α-functionalizations of ketones by the use of chiral iodine(iii) compounds usually give low to modest enantiomeric excess (<70%).^64,106^

### Mechanism of ketone oxidation using iodine(iii)

**Scheme 8:**
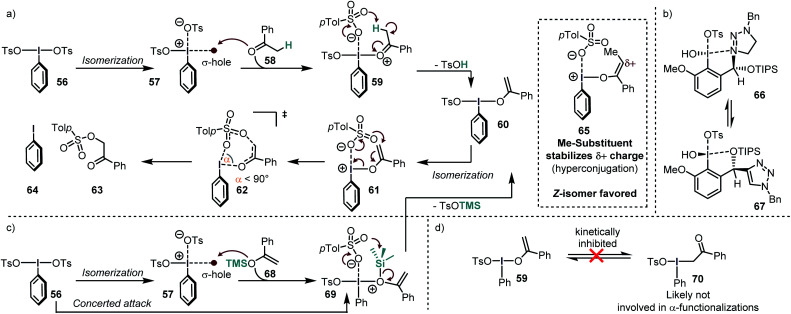
(a) Mechanism for the α-tosylation of acetophenone. (b) A novel “omnipotent” iodine(iii) compounds enables high enantioselectivities in several reactions. (c) Reasonable mechanism for the α-tosylation of the silyl enolether of acetophenone. (d) Calculated high barrier of O–C isomerization of the iodine(iii)–enol intermediate.

Legault and Beaulieu conducted a computational study of the α-tosyloxylation of acetophenones to find a rational for the low stereoinduction (Scheme 8a).^64^ According to their study, the reaction proceeds *via* initial activation of the iodine(iii) reagent PhI(OH)OTs by an acid to form PhI(OTs)_2_. In order to further react, this compound must isomerize to an energetically high lying species, in which the two electron-withdrawing groups are in *cis*-relationship (**57**). This requirement has been also found in other calculations for similar systems.^63^ In this isomer, the TsO–ligand *trans* to the arene is only loosely bound, so that the species can also be described as an intimate ion pair.

Due to this partial departure, it is possible that the σ-hole become sufficiently strong to act as a Lewis acid on the ketone so that the adduct **59** is formed. The high acidity of the coordinated ketone leads to deprotonation, which releases TsOH and the enol-species **60**. Although this compound is not positively charged, it is commonly named “enolonium species” due to its umpolung reactivity. Again, an isomerization to the energetically high lying isomer **61** takes place, which decomposes to the products *via* a seven-membered transition state (**62**). Although the ring size of the transition state is rather large, a narrow O–I–O angle (*α* < 90°) was calculated, deviating considerably from the heptagonal angle (*ca.* 128.5°). This narrow angle has also been observed in calculations of other transition states in I(iii) reductive elimination reactions proceeding through 6-,^116^ or 5-membered rings.^117,118^ Another important structural feature is that the I–O bond is almost perpendicular to the enol moiety. The last reaction step has been found to be the rate- and stereochemistry-determining event in the overall reaction, despite the small reaction barrier from **61** to **62** (*ca*. 3 kcal mol^−1^).

Most of the energy is required for the enolization and for the formation of the *cis* isomer (**61**). The overall reaction barrier was significantly lowered when an α-substituted acetophenone was used. The authors ascribe this stabilization to hyperconjugation into the electron-poor enol-species. Moreover, they found that the *Z*-enol species (**65**) reacted much faster than the corresponding *E*-isomer. They furthermore concluded that the low stereoinduction in the tosyloxylation reactions of ketones is due to the extended distance between the forming stereogenic center and the iodoarene. This issue has been tackled systematically by the introduction of triazole-substituted iodine(iii) compounds which convey their stereogenic information through extensive secondary bond interactions (most likely intramolecular I–N/I–O interactions – Scheme 8b).^119,120^ The catalytic tosyloxylation of propiophenone, for instance, was accomplished with 88% ee in this manner.

### Cross-nucleophile coupling involving enol-biased ketone derivatives

Because enolization is a rather unfavorable process but crucial for the reaction with iodine(iii) species, ketones which have a biased preference for the enol-tautomer are popular substrates for this kind of chemistry. In the following lines we will elaborate on the most commonly used ketone derivatives.

#### (a) Enol ethers, vinyl azides and metal enolates

Enol ethers, vinyl (pseudo)halides and enamines are very commonly employed in oxidations with iodine(iii) as the oxidant. Their use has been reviewed by Lauriers and Legault.^26^

Silyl enolether presumably attack through their oxygen atom (in a concerted or stepwise manner) and lose the electrofuge (TMS^+^) to form **60** from **69** (Scheme 8a and c). Although there are also contradicting reports (*vide infra*),^121^ this is in accordance with the NMR data provided by the group of Szpilman, where only the enol–iodine form (**59**) was detected when silyl enolethers were used^122^ and with the fact that the C–O-isomerization is kinetically inhibited for such systems (Scheme 8d).^64,118^

**Scheme 9:**
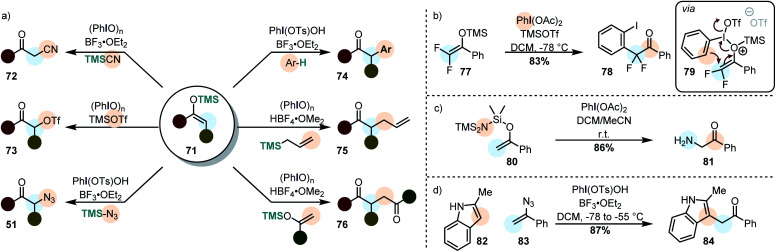
(a) Cross-nucleophile coupling of silyl enolethers. (b) Iodonio–Claisen rearrangement in a difluorosilyl enolether leading to β-functionalization of a cyclic silyl enolether. (c) Cross nucleophile coupling of silyl enolether with the nucleophile attached to the nucleofuge. (d) A vinyl azide reacts similarly in a cross-nucleophile coupling.

By using silyl enolethers as the starting materials, the electrophilic enol species **60** can be generated under mild reaction conditions (due to the enhanced reactivity towards electrophiles). This opens the possibility of using rather weak secondary nucleophiles for the cross-nucleophilic coupling (Scheme 9a).

It was already demonstrated in the 1980's that electrophilic enol species, can be attacked by other silyl enolethers (in homo-^123,124^ and cross-coupling reactions^125,126^ – **76**), allyl silanes (**75**) and even some plain alkenes.^125^ Moreover, it was demonstrated that α-trifloxy ketones (**73**) can be synthesized and isolated by this approach, showcasing the carbocationic character of iodine(iii)-mediated transformations.^127,128^ In more recent years, α-arylation (mainly with electron-rich arenes – **74**),^129,130^ azidation (**51**)^130,131^ and cyanation (**72**)^132,133^ have been accomplished. The cyanation is however rather limited to unsubstituted silyl enolethers. Moreover, α-arylation may become more general when diaryliodonium salts which favor arylation when electron-poor arenes are used.^134,135^ This is due to the fact that diaryliodonium salts operate *via* an alternative mechanism in which a reductive elimination takes place.^118^ As a matter of fact, very electron-rich arenes are so unreactive, that they can be used as “dummy-ligands” on the iodine atom of unsymmetrical diaryliodonium salts.^136,137^

In other approaches, the nucleophile was attached to the silane moiety during the starting material synthesis.^138^ Catalytic cross-nucleophile couplings with silyl enolethers are typically challenging due to prevailing background reactions. However, this can be overcome, even in an enantioselective fashion, by employing acetyl enol ethers.^139,140^ Contrary to silyl enolethers, these substrates are believed to attack the iodine(iii) compound from the carbon terminus.^140^

Very recently it was demonstrated that geminal difluorinated silyl enolethers (*cf.***77**) do not react with external nucleophiles. Instead they undergo a polarized [3,3]-sigmatropic rearrangement to give α-arylated ketones (Scheme 9b).^121^ The two fluorine atoms were made accountable for this unusual reactivity: backed by DFT-calculations, the authors proposed that the two electronegative atoms lead to a preferential *O*-attack of the silyl enolether on the activated iodine(iii) species. This explanation stands however in contrast to the experimental results by Szpilman *et al.* who showed that also simple silyl enolethers have a large preferences for the *O*-attack of the substrate (*vide supra*).^122^ Interesting is also that the electrofuge (TMS^+^) was calculated to leave the substrate after the oxidation event (the sigmatropic rearrangement).

Wirth *et al.* developed an interesting strategy in which the nucleophile is already attached to the silyl enol ether (Scheme 9c).^138^ This allows for the very efficient preparation of α-functionalized ketones by using *O*-,*N*- and *C*-nucleophiles.

Vinyl azides (*cf.***83**) can be similarly used, as shown very recently by Szpilman *et al.* (Scheme 9d). They react with the electrophilic iodine(iii) compound through the nitrogen atom adjacent to the olefin, forming an azido enolonium species. This highly reactive species adds nucleophiles at the α-position, extruding nitrogen to yield α-functionalized ketones.^141^ Vinyl chlorides and vinyl bromides react to give similar products, although probably *via* a different mechanism.^142^ When vinyl azides are treated with a mixture of PIDA and Py·9HF, on the other hand, the azide moiety undergoes an interesting 1,2 migration to yield 1,1-difluoro-2-azides.^143^

Although the majority of oxidations with iodine(iii) compounds is done under acidic conditions, *in situ* generated metal enolates can also productively be engaged.^98^

#### (b) Active methylene/methine compounds

**Scheme 10:**
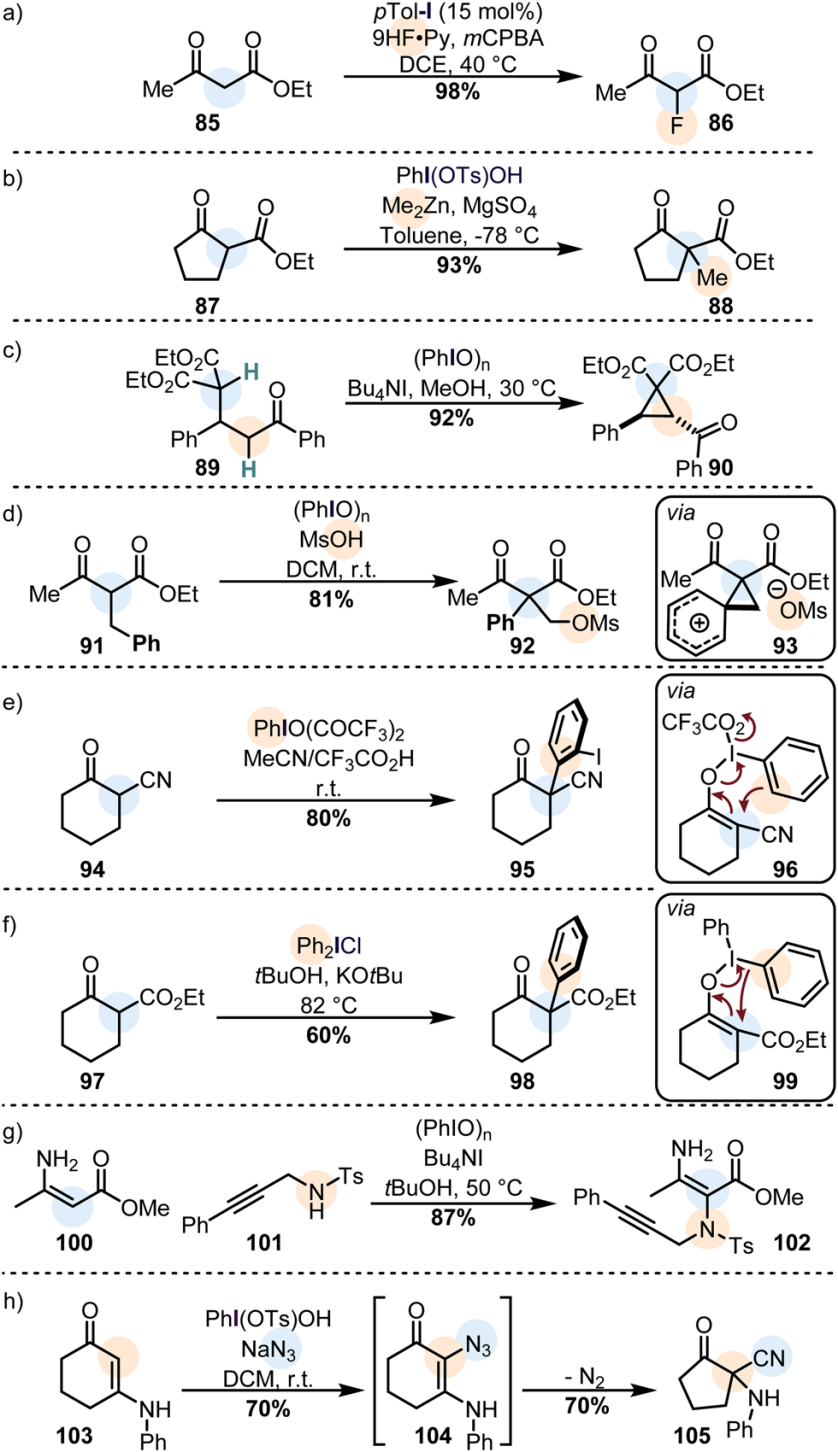
β-Dicarbonyls are often used in iodine(iii) oxidations due to their large enol content. (a) α-Fluorination of a β-ketoester. (b) Cross-nucleophile coupling with an organometallic nucleophile. (c) Intramolecular cyclopropanation by oxidation of a β-diester. (d) Carbocationic phenyl migration in an oxidized β-ketoester. (e) Arylation *via* an iodonio-Claisen rearrangement (f) arylation *via* a remote reductive elimination. (g) An enaminone reacts with iodine(iii) and a nucleophilic azide. (h) An enaminone reacts with an azide anion with a subsequent rearrangement.

Active methylene compounds are widely used as reactive substrates for iodine(iii) oxidations due to their biased enol content. For instance, β-ketoesters can be fluorinated catalytically (Scheme 10a),^144^ or stoichiometrically.^145,146^

Alkylations of similar substrates can be achieved by using a combination of Koser's reagent and a dialkylzinc species (Scheme 10b),^147^ and Michael adducts can be oxidatively cyclized to form cyclopropanes by using PhIO in methanol (Scheme 10c).^148^ Similarly, the oxidation of β-aryl active methylene compounds may lead to a 1,2-aryl migration with subsequent capture of the transient phenonium ion by the secondary nucleophile (Scheme 10d).^149^ Another interesting example is the use of iodine(iii) on cyclic active methine substrates. In these cases, the enolonium species **96** (*cf.* compound **60**) does not react with one of the ligands: instead, the aromatic ring of the iodine(iii) reagent reacts as the nucleophile in a polarized [3,3]-sigmatropic rearrangement to furnish the α-arylated product (Scheme 10e).^116,150^ Diaryliodonium salts react with similar substrates (usually under basic conditions) but in those cases the aryl moiety is transferred with loss of ArI (Scheme 10f).^118,151^

Vinylogous amides are another privileged class of primary nucleophiles for cross-nucleophilic coupling reactions mediated by iodine(iii). Propargylic amines, for instance, can be coupled efficiently to ester-substituted enamines *via* the oxidation of the latter (Scheme 10g).^152^ It was recently shown that cyclic enaminones can be similarly coupled to azides (Scheme 10h). The α-azido enaminone intermediate **104** rearranges to cyclopentanone **105** under extrusion of nitrogen.^153^ The generated products can be further rearranged by the action of iodine(iii).^154^

#### (c) Michael addition/oxidation tandem on enones

An alternative strategy to circumvent energetically disfavoured enolization consists in the generation of enols/enolates by 1,4-addition to α,β-unsaturated compounds. The *in situ* generated enol/enolate subsequently react with electrophilic iodine(iii) oxidants to yield α,β-functionalized products.

**Scheme 11:**
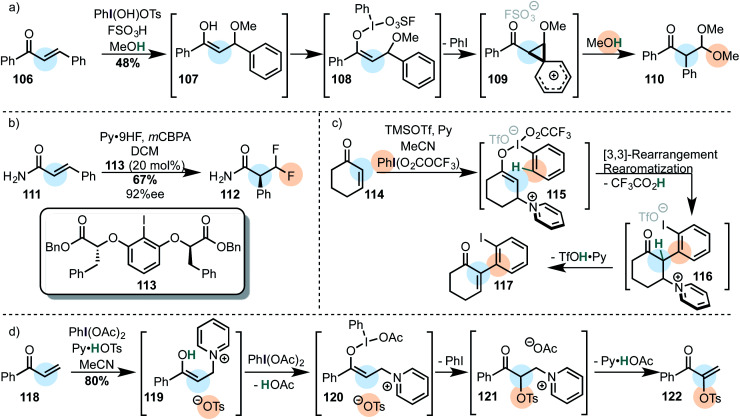
Michael acceptors as substrates in 1,4-addition/iodine(iii) oxidation tandem reactions. (a) Classical example with methanol as the secondary nucleophile. (b) Catalytic enantioselective difluorination *via* an iodine(iii)-mediated phenyl migration. (c) The 1,4-addition of the bulky pyridine blocks the enolonium species from nucleophilic attack and instead enables an iodonio-Claisen rearrangement. (d) Umpolung of the Morita–Baylis–Hillman reaction.

β-Phenyl-α,β-unsaturated ketones are known to rearrange *via* a Michael addition/oxidation/1,2-phenyl shift domino since the 1980's and were used to afford acetals (Scheme 11a).^75^‡‡If the conditions are varied, the acetal product (**110**) may be further oxidized to yield the α-aryl–α-keto carbonyl compound.^163^

An enantioselective version (by use of a stoichiometric iodine(iii) oxidant) was published in 2013.^155^ This approach was further developed three years later in a catalytic asymmetric difluorination reaction of α,β-unsaturated carbonyl compounds. In this case, a geminal β-difluoride is generated with high enantioselectivity (Scheme 11b).^156^

Very recently, cyclic enones were shown to react *via* a Michael addition-oxidation sequence in which a pyridine first adds to the enone in order to form an enolate which effectively reacts with the iodine(iii) oxidant (Scheme 11c).^157^ This is a smart variation of the reaction discovered by Shafir (Scheme 10e and f). Because the pyridinium blocks the enolonium species for a conventional nucleophilic attack, a [3,3]-sigmatropic rearrangement takes place instead, furnishing the α-arylated species **116**. Elimination of the pyridine finally yields the *O*-iodostyrene product **117**. A few months later, Szpilman *et al.* showed that a similar enolonium species intermediate can be used in a direct umpolung of the Morita–Baylis–Hillman reaction (Scheme 11d). The suspected intermediate **121** has been observed by NMR *in situ*.

## Conclusions and outlook

With this perspective article, an overview of important developments in the field of iodine(iii) chemistry was provided with a focus on (a) recently discovered structural features of “hypervalent” iodine compounds and (b) their use for oxidative cross-nucleophile coupling reactions.

It is apparent that the discussed properties of iodine(iii) compounds represent a potential leverage for the discovery of novel reactivity. This is also showcased by the ongoing interest in the development of new iodine(iii) and iodine(v) compounds.^119,158–161^ The use of ketones and their derivatives is particularly interesting, because the 2e^−^ oxidation gives rise to an α-carbocationic ketone synthon, which prone to undergo carbocationic rearrangements. This will continue to inspire chemists to design new sequences and serve as an engine for serendipitous discovery.

## Conflicts of interest

There are no conflicts to declare.
